# Negative charge and membrane-tethered viral 3B cooperate to recruit viral RNA dependent RNA polymerase 3D^**pol**^

**DOI:** 10.1038/s41598-017-17621-6

**Published:** 2017-12-11

**Authors:** Anna Dubankova, Jana Humpolickova, Martin Klima, Evzen Boura

**Affiliations:** Institute of Organic Chemistry and Biochemistry of the Czech Academy of Sciences, Prague, Czech Republic

## Abstract

Most single stranded plus RNA viruses hijack phosphatidylinositol 4-kinases (PI4Ks) to generate membranes highly enriched in phosphatidylinositol 4-phosphate (PI4P). These membranous compartments known as webs, replication factories or replication organelles are essential for viral replication because they provide protection from the innate intracellular immune response while serving as platforms for viral replication. Using purified recombinant proteins and biomimetic model membranes we show that the nonstructural viral 3A protein is sufficient to promote membrane hyper-phosphorylation given the proper intracellular cofactors (PI4KB and ACBD3). However, our bio-mimetic *in vitro* reconstitution assay revealed that rather than the presence of PI4P specifically, negative charge alone is sufficient for the recruitment of 3D^pol^ enzymes to the surface of the lipid bilayer. Additionally, we show that membrane tethered viral 3B protein (also known as Vpg) works in combination with the negative charge to increase the efficiency of membrane recruitment of 3D^pol^.

## Introduction

Space in the capsids of small viruses is limited and small viruses do not encode every enzymatic activity required for their replication. Single stranded plus RNA (+RNA) viruses replicate at replication organelles (also known as replication factories or membranous webs) which provide an optimal replication environment and also protection from innate immunity^1^. Membranous webs are highly enriched in the signaling lipid PI4P (phosphatidylinositol 4-phosphate), yet +RNA viruses do not encode phosphatidylinositol 4-kinases (PI4Ks). Instead, they hijack a human enzyme, either PI4KA or PI4KB (also called PI4K IIIα or PI4K IIIβ)^2–7^. The other two human PI4K isoforms, PI4K2A and PI4K2B (also known as PI4K IIα or PI4K IIβ), are palmitoylated proteins^8^, and this posttranslational modification possibly renders them a more difficult target for viruses to recruit. It is also possible that the different subcellular localization of different PI4K enzymes is the reason why PI4KB and PI4KA are hijacked by viruses and PI4K2A and PI4K2B are not^9^. However, although the subcellular localization of PI4K2A and PI4KB is similar (both are mainly Golgi localized), only PI4KB has been reported to be hijacked by viruses. In addition, although the subcellular localization of PI4KA and PI4KB is different (plasma membrane vs Golgi) both enzymes are used by HCV (the preference depends on the HCV genotype).

PI4Ks have been characterized extensively because they are essential host factors for many + RNA viruses^9^. Crystal structures of all PI4K isoforms except PI4KA are available^10–12^, their complexes with binding partners Rab11, ACBD3 and 14-3-3 were structurally characterized^10,13–15^ and potent and extremely selective inhibitors that exert antiviral activity have been developed^16–19^. PI4Ks are hijacked by viruses either directly or indirectly. Hepatitis C virus (HCV) uses a direct mechanism: its nonstructural NS5A protein directly binds and recruits PI4KA^3^. Similarly, the nonstructural 3A protein from the encephalomyocarditis virus (EMCV, genus Cardiovirus) interacts directly with PI4KA^20^. In contrast, most picornaviruses use an indirect mechanism with several variations among different members of the *Picornaviridae* family. ACBD3 (acyl-CoA-binding domain-containing protein-3) was recently shown to form a strong complex with PI4KB, activating its lipid kinase activity^13,21^. Nonstructural 3A proteins from several enteroviruses including poliovirus (PV) and coxsackievirus B3 (CVB3) can interact with both guanine nucleotide exchange factor-1 (GBF1)^22,23^ and ACBD3 simultaneously^5,24^. Distinct, nonstructural 3A proteins from kobuviruses such as Aichi virus use nearly all their residues to interact with ACBD3^5,24,25^. In addition, genetic ablation of ACBD3 prevents the recruitment of PI4KB to Aichi virus replication sites^21^. It is important to mention that the picornavirus proteins arise from proteolytical processing of the viral polyprotein in such a way that various stable intermediates such as 3AB or 3CD are generated and that only membrane anchored 3AB is a substrate for the viral 3CD and 3 C protease^26^ and also that the membrane composition plays a regulatory role^27^. The poliovirus 3AB protein was previously suggested to anchor the RNA replication complex to the membrane^28^ through the C-terminal hydrophobic part of the 3A protein^29^ and the 3AB fusion protein was also previously shown to activate the poliovirus 3D^pol^ enzyme^30^. The 3B protein (also known as VPg from viral protein genome linked) arises from the 3AB precursor by cleavage by the proteinase 3CD^pro^ and serves as a primer for the 3D^pol^ enzyme^31^.

The reason membranous webs are highly enriched in PI4P is still poorly understood. Picornaviral polymerases are active on their own (without any protein co-factor although a primer, which is the 3B protein *in vivo* but can be both 3B or nucleic acid *in vitro* must be present). However, upon assembly of the replication complex their processivity is believed to significantly increase, more details can be found in recent review by OB Peersen^32^. Another intriguing feature of PI4P is that it can be exchanged for other lipids such as cholesterol^33–35^ or phosphatidylserine (PS)^36^ against concentration gradients because PI4P hydrolysis at the target membrane generates energy^37^. Indeed, production of PI4P to modify the cholesterol content of membranous webs was demonstrated for the encephalomyocarditis virus (EMCV)^20^. Another possible function of PI4P could be direct recruitment of viral effector proteins to the replication sites. PI4P binding proteins are well described in several pathogenic bacteria such as the SidC and SidM proteins from Legionella^38^. 3D^pol^ could be such a viral factor. If 3D^pol^ could bind PI4P it would be recruited to replication sites in a PI4P-dependent manner. This mechanism was reported for poliovirus (PV), and PI4P-mediated 3D^pol^ recruitment was proposed as a mechanism for picornaviral and flaviviral replication^39^. However, whether PI4P hyper-production is sufficient to recruit the polymerase to the surface of the lipid bilayer is not clear.

Here we sought to directly test the hypothesis that 3D^pol^ RNA polymerase is recruited to hyper-phosphorylated membranes using a clearly defined *in vitro* system. We reconstituted the initial generation of membranous web using purified recombinant proteins of the human Aichi virus and the biomimetic giant unilamellar vesicle (GUV) system. GUVs are very large vesicles comparable in size to human cells making them ideal for confocal microscopy. GUVs can also be filled with a sucrose solution which makes them heavier than the surrounding buffer, and as a result they do not move but instead sit at the bottom of the chamber where they are imaged. Additionally, GUVs can be prepared from almost any lipid mixture such that their lipid composition resembles that of the specific organelle they are mimicking (here, we use a mixture resembling the Golgi and viral replication organelles). For these reasons GUVs are used to reconstitute and thereby, gain molecular insight into biologically important processes that involve membranes. For instance, the ESCRT (endosomal sorting complex required for transport) complex catalyzes membrane scission^40–42^ and GUVs were used to understand this reaction *in vitro* ^43^. GUVs were also used to elucidate the mechanism of assembly and ESCRT recruitment by HIV Gag^44,45^, the clathrin assembly on the surface of GUVs^46^, and the function of Endophilin-A2 in endocytosis^47^. Additional examples and practical applications can be found in a recent book on model membranes^48^.

We chose the human Aichi virus as a model organism because the interaction of its nonstructural 3A protein with host ACBD3 is well described^49,50^ and reported to be required for the recruitment of PI4KB to the viral replication sites *in vivo* ^21^. We demonstrate that the 3A protein is sufficient to facilitate membrane PI-phosphorylation when the appropriate cellular cofactors (ACBD3 and PI4KB) are available. However, although PI4P production alone did not lead to efficient membrane recruitment of 3D^pol^, the situation changed when membrane-tethered 3B protein was present. We demonstrate that not only the negatively charged PI4P but another negatively charged lipid, such as PS, can cooperate with membrane-anchored 3B to recruit the 3D^pol^ enzyme.

## Results

### 3A efficiently recruits ACBD3 to model membranes

**Figure 1:**
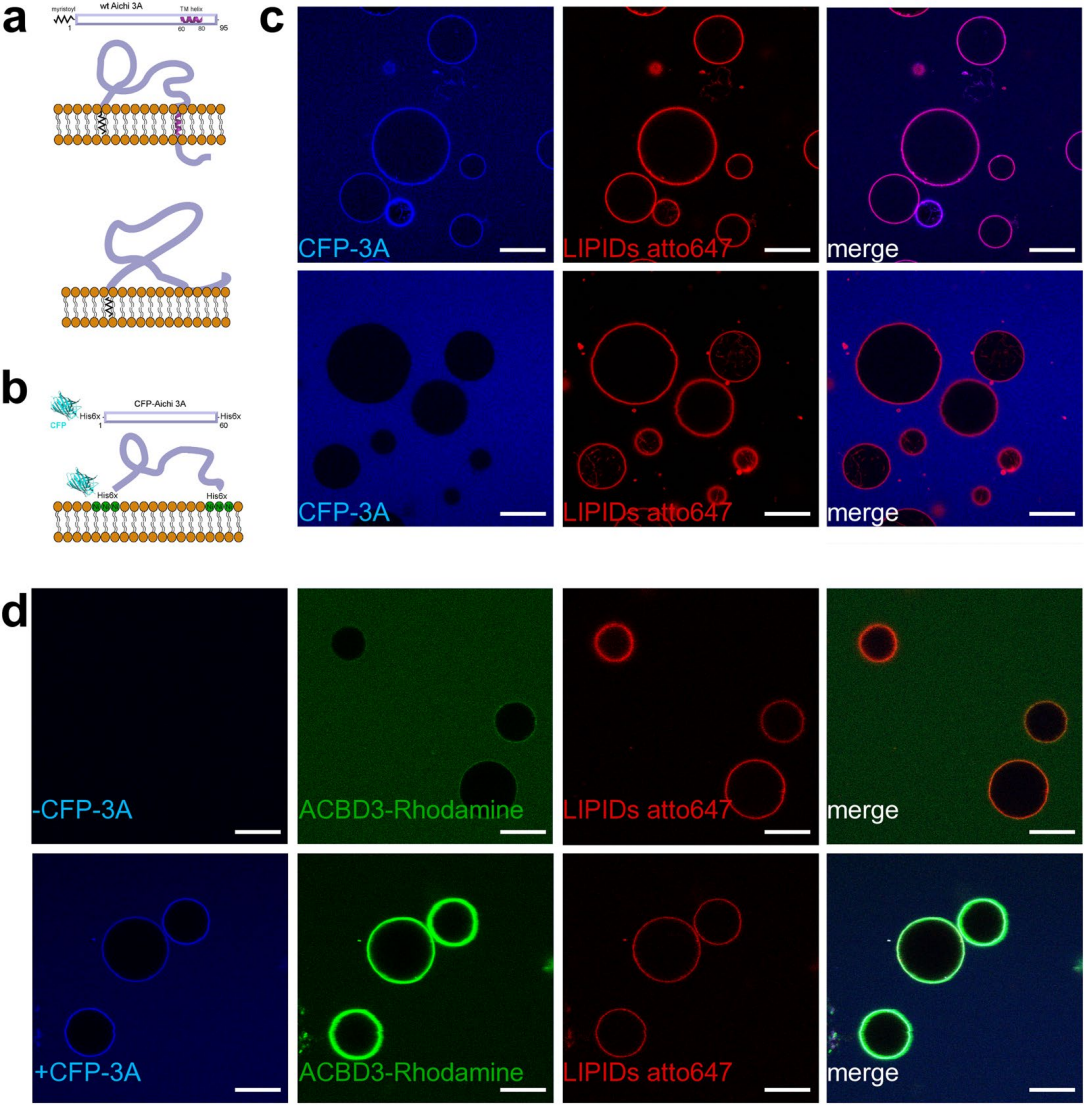
Aichi virus 3A protein on the membrane. (**A**) Schematic representation of the wild type 3A protein. The two possible topologies of the wild type 3A protein are shown. Upper panel – the C-terminal hydrophobic stretch is depicted as a transmembrane helix. Lower panel – the C-terminal hydrophobic stretch is depicted as semi-buried in the lipid bilayer. (**B**) Schematic representation of the mCerulean - 3A fusion protein (named CFP-3A). CFP-3A contains two 6xHis tags; one between the CFP and its N-terminus and one at the C-terminus. These two His tags can be used to attach the CFP-3A protein to a membrane containing DGS-NTA(Ni) (a lipid that has Ni^2+^ bound to its headgroup). (**C**) CFP-3A bound to GUVs. Upper panel: 250 nM CFP-3A was added to GUVs containing 5% of DGS-NTA(Ni) and ATTO647N-DOPE (0.1 mol %). Lower panel: as above but DGS-NTA(Ni) was replaced by POPC. The CFP-3A signal is in blue and the ATTO647 signal in red. Representative image of three independent experiments. Scale bar = 20 µm. (**D**) Aichi virus 3A protein efficiently recruits ACBD3 to the membrane. Rhodamine labeled ACBD3 (250 nM) was incubated with GUVs without 3A protein (upper panel) or with 3A protein (250 nM, lower panel). The CFP-3A signal is in blue, the ACBD3-rhodamine signal is in green, and ATTO647 labeled lipids in in red. Representative image of three independent experiments. Scale bar = 20 µm.

In order to faciliate the recruitment of ACBD3 to model membranes, we engineered a biomimetic recombinant 3A viral protein. The 3A protein is myristoylated at the N-terminal glycine residue^24^ and also contains a hydrophobic region that every algorithm tested (CCTOP, HMMTOP, MemBrain, Memsat, Octopus, Philius, Phobius, Pro, Prodiv, Scampi, ScampiMsa, and TMHMM) predicts as a transmembrane helix that could anchor 3A protein in the lipid bilayer (Fig. 1A upper panel). This is also supported by our all-atom molecular dynamics simulations of the 3A:GOLD domain complex on the surface of the lipid bilayer^49^. Alternatively, it was suggested for the poliovirus 3AB protein that this hydrophobic region is semi-buried in the lipid bilayer^51^ as depicted in the lower panel of Fig. 1A. We fused CFP and a 6xHis tag to the N-terminus of the 3A protein and we replaced the transmembrane helix with another 6xHis tag. The resulting CFP-His_6_-3A-His_6_, is tethered by both its N-terminus and C-terminus to a Ni^2+^ containing membrane in a similar fashion to that of the wild type protein (Fig. 1B). A pilot experiment revealed that the CFP-His_6_-3A-His_6_ could be tethered to artificial membranes that contain a nickel cation-bound lipid such as the DGS-NTA(Ni) (Fig. 1C, upper panel) but not to control membranes (Fig. 1C, lower panel).

ACBD3 is a Golgi resident scaffolding protein^52^ that has been reported to interact with 3A proteins from several +RNA viruses including the Aichi virus^5,21,24,25,53^. We sought to directly test this interaction *in vitro* and in the context of a membrane. Recombinant ACBD3 labeled with rhodamine was only weakly localized to the membrane at 250 nM concentration (Fig. 1D, upper panel). However, when membranes harbored viral 3A protein, ACBD3 was efficiently recruited to these membranes (Fig. 1D, lower panel).

### 3A, ACBD3, and PI4KB form a protein complex on model membranes

**Figure 2:**
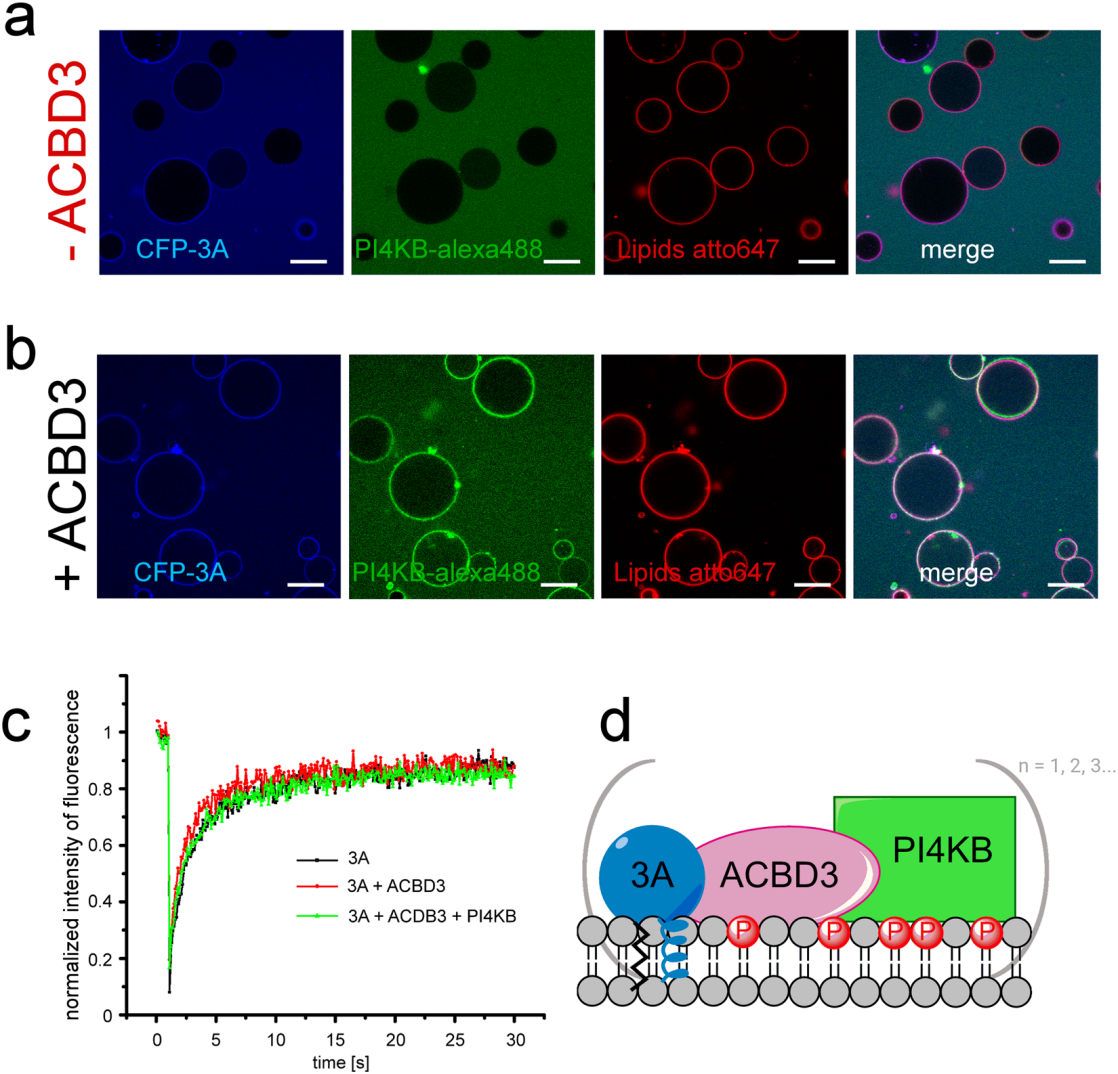
Aichi virus 3A protein recruits the PI4KB kinase via ACBD3. (**A**) 3A protein does not recruit PI4KB directly to GUVs. 250 nM CFP-3A and PI4KB labeled by Alexa488 were added to GUVs containing 5% of DGS-NTA(Ni) and ATTO647N-DOPE (0.1 mol %). The CFP-3A signal is in blue, the PI4KB-Alexa488 signal is in green and the ATTO647 signal is in red. Representative image of three independent experiments. Scale bar = 20 µm. (**B**) PI4KB is recruited to membranes when ACBD3 is present. CFP-3A, Alexa488 labeled PI4KB and unlabeled ACBD3 (250 nM each) were added to GUVs containing 5% DGS-NTA(Ni) and ATTO647N-DOPE (0.1 mol %). The CFP-3A signal is in blue, the PI4KB-Alexa488 signal in green and the ATTO647 signal is in red. Representative image of three independent experiments. Scale bar = 20 µm. (**C**) FRAP analysis of the 3A:ACBD3:PI4KB protein complex. A small cross-section of a GUV membrane was intensively bleached by a 405 nm laser and fluorescence recovery after photobleaching (FRAP) of CFP-3A was measured. (**D**) Schematic representation of the 3A:ACBD3:PI4KB protein complex.

PI4KB is a lipid kinase that must be recruited to membranes to function properly. The interaction of ACBD3 with PI4KB was previously found to have a dissociation constant of 320 nM, and ACBD3 can recruit PI4KB to membranes when ACBD3 is artificially tethered to the membrane surface^13^. We therefore sought to test if 3A recruited ACBD3 was capable of further recruiting PI4KB to form a larger 3A:ACBD3:PI4KB complex on the membrane. PI4KB is efficiently recruited to model membranes decorated with CFP-3A and ACBD3 (Fig. 2A,B). Next we aimed to elucidate if 3A:ACBD3:PI4KB forms oligomers on the surface of the lipid bilayer. We used fluorescent recovery after photobleaching (FRAP) in which large protein clusters would show significantly slower fluorescence recovery than individual protein complexes^54^. We compared the FRAP of the CFP-3A, CFP-3A:ACBD3, and CFP-3A:ACBD3:PI4KB (Fig. 2C) and found no statistically significant differences. Based on the fast FRAP we assume that the 3A:ACBD3:PI4KB protein complex forms a heterotrimer or smaller oligomers on the surface of the membrane but not large assemblies such as lattices that cannot exhibit fast FRAP (Fig. 2D).

### PI4KB is highly active as part of the 3A:ACBD3:PI4KB protein complex

**Figure 3:**
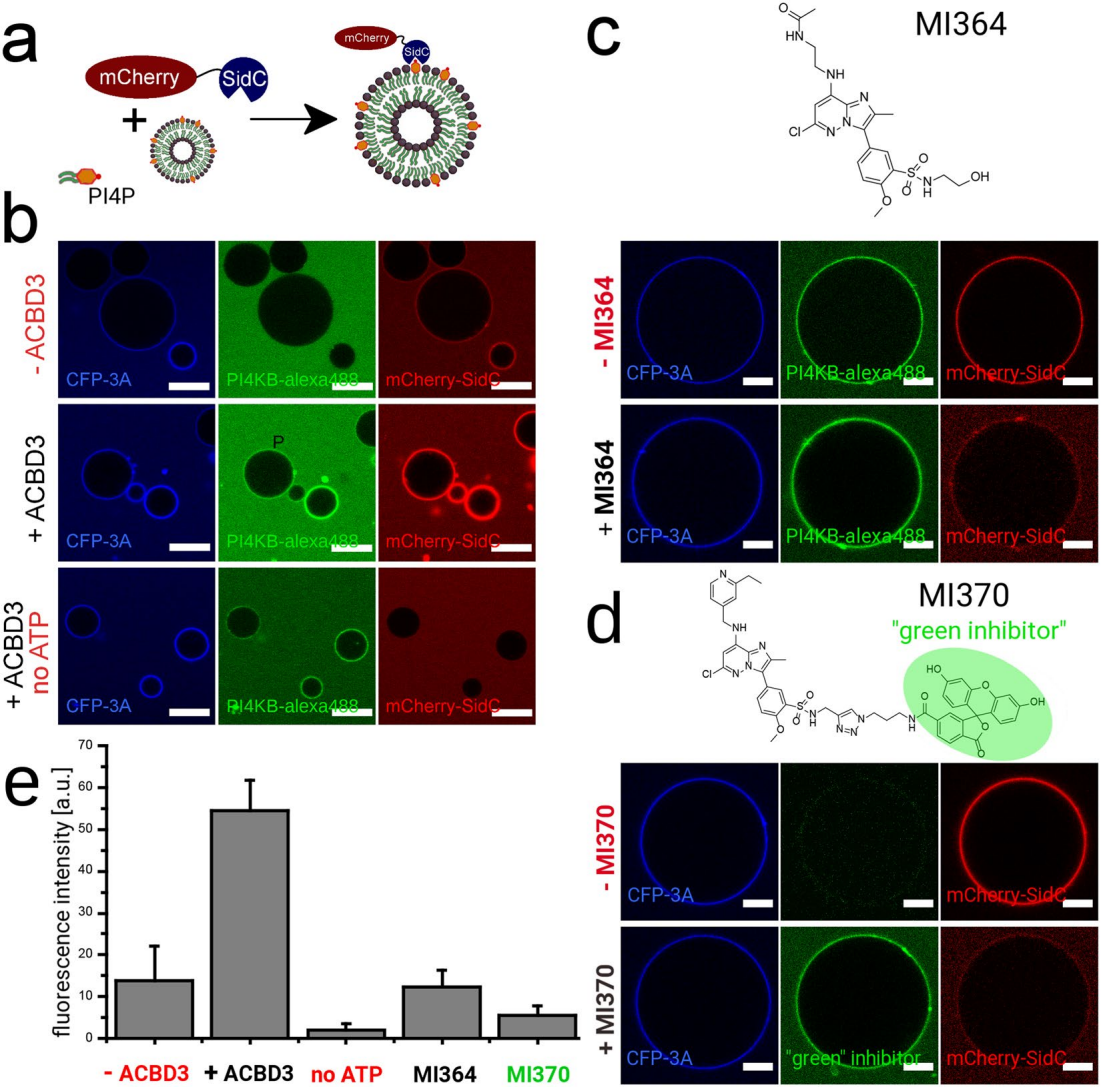
PI4KB is activated in the 3A:ACBD3:PI4KB protein complex. (**A**) Scheme of the experiment. A PI4P binding domain from the Legionella pneumonia SidC protein that binds to PI4P with nanomolar affinity was fused to mCherry and used as a fluorescent PI4P biosensor. (**B**) Production of PI4P on the surface of GUVs. Upper panel: GUVs decorated with the viral 3A-CFP protein (250 nM) were incubated with Alexa488 labeled PI4KB (250 nM) and SidC-mCherry (100 nM) and imaged using a confocal microscope. Middle panel: As above but also ACBD3 (250 nM) was also added. Lower panel: As the middle panel but no ATP was added. The CFP-3A signal is in blue, the PI4KB-Alexa488 signal in green and the mCherry-SidC signal is in red. Representative image of three independent experiments. Scale bar = 20 µm. (**C**) Inhibition by the compound MI364. On the top is the chemical structure of MI364. On the bottom is a representative image of GUVs phosphorylated by Alexa488 labeled PI4KB (upper panel: without MI364, lower panel: with 5 µM MI364). The CFP-3A signal is in blue, the PI4KB-Alexa488 signal in green and the mCherry-SidC signal is in red. Representative image of three independent experiments. Scale bar = 10 µm. (**D**) Inhibition by the fluorescent compound MI370. On the top is the chemical structure of MI370 (the fluorescent part in highlighted in green). On the bottom is a representative image of GUVs phosphorylated by unlabeled PI4KB (upper panel: without MI370, lower panel: with 5 µM MI370). The CFP-3A signal is in blue, the MI370 signal in green and the mCherry-SidC signal is in red. Representative image of three independent experiments. Scale bar = 10 µm. (**E**) Quantification of PI4P production. Quantification of the intensity of fluorescence of the SidC-mCherry PI4P biosensor on the surface of GUVs was. Standard deviations are based on three independent experiments.

Only membrane-tethered ACBD3 activates the PI4KB enzyme^13^. It is likely that viruses use their 3A protein to tether the ACBD3 protein to the membrane in order to activate PI4KB^21^. The 3A protein is perfectly suited for this job because it is membrane tethered and tightly binds ACBD3. To test this hypothesis *in vitro* we used a SidC fluorescent PI4P biosensor (PI4P binding domain of SidC from Legionella pneumonia fused to mCherry)^38^ to detect the relative amount of PI4P synthesized on the surface of GUVs (Fig. 3A). GUVs were first decorated with the viral CFP-3A protein and then incubated with PI4KB alone or with ACBD3 and PI4KB (Fig. 3B upper and middle panels). The control consisted of a mixture of all proteins but was devoid of ATP (Fig. 3B lower panel). The efficiency of membrane phosphorylation (the conversion of PI to PI4P) was determined from the biosensor’s fluorescent signal on the surface of the membrane. Background levels of PI4KB lipid phosphorylation in the presence of only the 3A protein decorated GUVs were measured (Fig. 3B upper panel, Fig. 3C). However, the observed efficiency of the reaction in the presence of ACBD3 was far higher as judged by the bio-sensor recruitment (Fig. 3B middle panel). In the absence of ATP, no membrane phosphorylation was observed even when all the proteins were present (Fig. 3B lower panel), documenting the specificity of the system. As an additional control we performed the phosphorylation reaction in the presence of the specific PI4KB inhibitor MI364 (compound **23** in our recent publication^55^) and, as expected, we observed only limited recruitment of the mCherry-SidC biosensor to the GUVs (Fig. 3C). Furthermore, we performed an analogous experiment using unlabeled PI4KB and a fluorescent PI4KB inhibitor (compound **3** in our recent publication^56^). Again, we observed only limited recruitment of the mCherry-SidC biosensor. However, in this setting the fluorescent inhibitor could be used to visualize unlabeled PI4KB on the surface of the GUVs (Fig. 3D). Quantification of the fluorescence intensity revealed that the apparent activity of the 3A:ACBD3:PI4KB protein complex was roughly 4-fold higher than that of the PI4KB kinase alone in this system (Fig. 3E).

### PI4P and Aichi 3D^pol^ membrane recruitment

**Figure 4:**
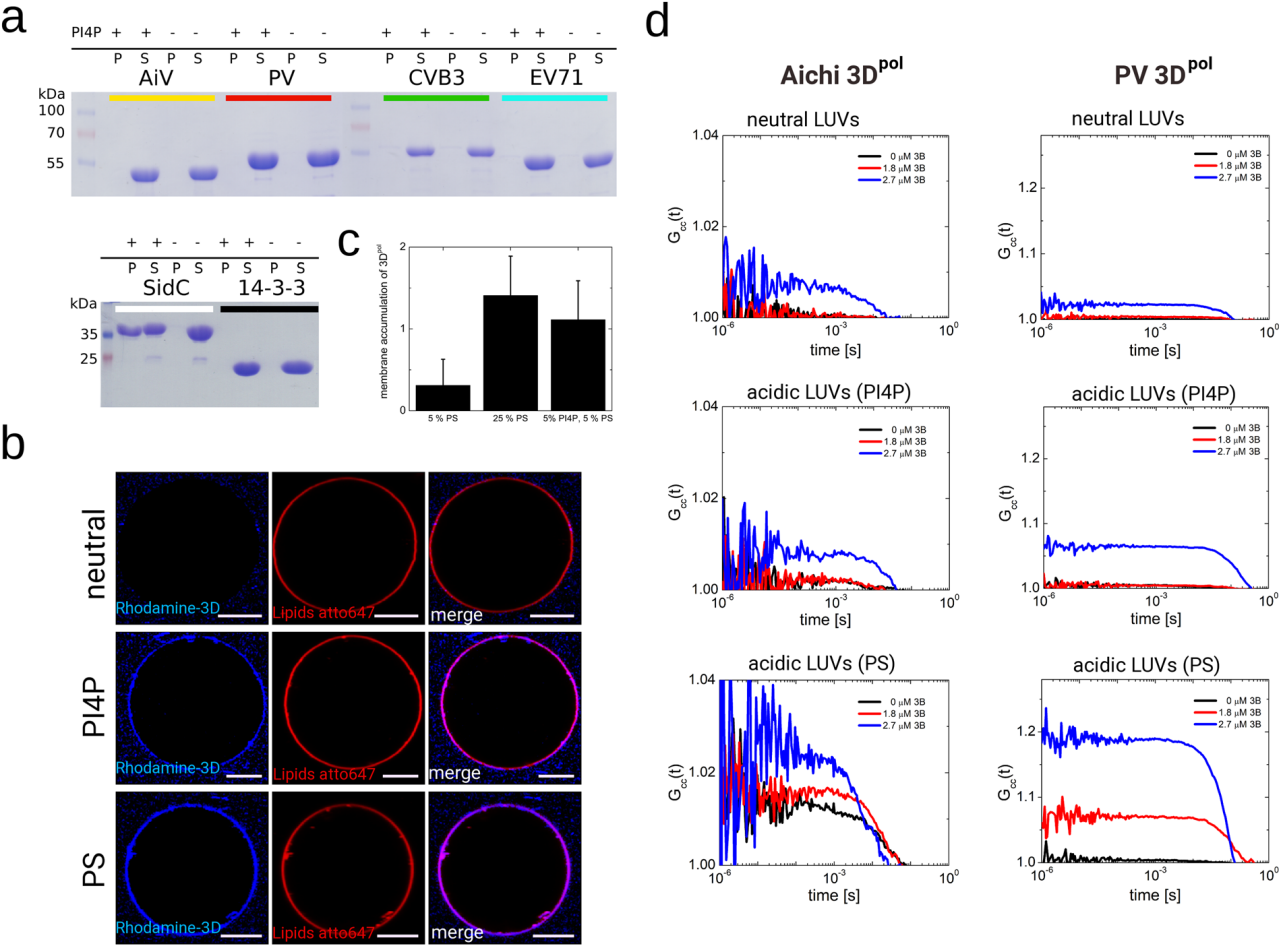
3D^pol^ enzymes efficiently bind acidic membranes only in the presence of membrane-tethered 3B. (**A**) Recombinant 3D^pol^ enzymes from several +RNA viruses bind PI4P poorly in a liposome pulldown assay. 3D^pol^ from the Aichi virus (AiV), Polio virus (PV), Coxsackie virus B3 (CVB3) and Enterovirus 71 (EV71) show no binding to PI4P liposomes at 60 µM concentration. SidC was used as a positive control and 14-3-3ζ protein as a negative control. Liposomes were centrifuged and the pellets (denoted P) and supernatants (denoted S) were analyzed using SDS PAGE. For the full length gels see the SI Fig. 2. (**B**) GUV recruitment assay. GUVs of different composition were used. Upper panel – neutral membrane (5% PS), middle panel – PI4P acidic membrane (5% PS + 5% PI4P), lower panel – PS acidic membrane. Aichi 3D^pol^ fluorescence signal in blue (left), membrane in red (middle) and merged image (right). A typical result of three independent experiments. Scale bar = 20 µm. (**C**) Quantification of Aichi 3D^pol^ binding to membranes of different composition. Composition dependent membrane accumulation of CFP-3D^pol^: excess of the fluorescence signal of CFP in the GUV pixels relative to the signal of unbound CFP-3D^pol^. The error bars indicate a 95% confidence interval. (**D**) Cros-scorrelation curves of CFP – 3D^pol^ and LUVs. FCS curves representing temporal cross-correlation of the CFP-3D^pol^ (Aichi 3D^pol^ on the left panel and PV 3D^pol^ on the right panel) and ATTO647N-DOPE labelled LUVs at various membrane lipid compositions (neutral, enriched by PI4P, and enriched by PS; see SI Table 1). The curves show the cooperative effect of the 3B peptide on 3D^pol^ membrane recruitment. The concentration of the Aichi or PV 3B peptide (attached to the membrane surface by His-tag – 18:1 DGS-NTA(Ni) interaction) was 0 µM (black), 1.8 µM (red), and 2.7 µM (blue).

The reconstitution of ACBD3 recruitment to model membranes by viral 3A, and the subsequent recruitment of the lipid kinase PI4KB made possible the analysis of the membranes binding properties of 3D^pol^. We used our *in vitro* GUV system to test PI4P mediated 3D^pol^ recruitment because this mechanism was suggested previously^39^. As shown above, the 3A:ACBD3:PI4KB complex efficiently phosphorylates GUVs to make PI4P (Fig. 3). However, we did not observe 3D^pol^ membranes recruitment even though the mCherry-SidC nanomolar PI4P binder biosensor was recruited (SI Fig. 1A). Similarly, 3D^pol^ failed to bind GUVs prepared with synthetic PI4P (SI Fig. 1B). We reasoned that the 3D^pol^:PI4P interaction might be weak, or that the Aichi 3D^pol^ is unique among 3D^pol^ enzymes in that it does not bind PI4P. Therefore, we tested several different 3D^pol^ enzymes (Aichi virus, PV, CVB3, EV71) in a liposome pulldown assay. However, no PI4P-mediated liposome binding of any of the polymerases was detected using this method (Fig. 4A).

Pulldown assays are not sensitive and a weak but biologically relevant interaction could be overlooked. Therefore, we performed a more sensitive experiment. GUVs were incubated with fluorescently (rhodamine) labeled Aichi 3D^pol^ at nanomolar concentration, which facilitates efficient binding due to the vast excess of lipids. In addition, the fluorescence image was taken using a low scanner speed (50 µs per pixel) to ensure a higher signal-to-noise ratio (Fig. 4B). We used GUVs with PI4P and two sets of controls: neutral GUVs in which PI4P was replaced with the neutral lipid PC and an acidic membrane where PI4P was replaced with the negatively charged PS lipid (we used four molecules of PS for every molecule of PI4P to create a highly acidic membrane without the PI4P lipid). We observed that 3D^pol^ does not bind to neutral GUVs (Fig. 4B upper panel). However, 3D^pol^ did bind the PI4P-containing GUVs (Fig. 4B middle panel) suggesting that the 3D^pol^ enzyme is, indeed, capable of binding PI4P containing membranes albeit with low affinity. Surprisingly, the 3D^pol^ enzyme could also bind PS-containing acidic GUVs (Fig. 4B lower panel). Quantification of membrane binding revealed that 3D^pol^ enzyme binds acidic GUVs without a preference for specific acidic lipids (Fig. 4C).

However, the extent of 3D^pol^ recruitment by model acidic membranes seems too low to support efficient viral replication within the host cell. The only other reported binding partner of any 3D^pol^ enzyme (besides the RNA) is the small 3B protein that serves as a primer. A portion of 3B in the infected cell exists as a membrane tethered 3AB fusion protein. Thus, we used our *in vitro* system to test whether membrane-tethered 3B and the acidic membrane can cooperate to recruit 3D^pol^ enzymes. We could not use GUVs for this experiment because the reported dissociation constants between 3B and 3D are in the tens of micromolar range and it is not possible to use micromolar lipid concentrations in the case of GUVs. We instead used large unilamellar vesicles (LUVs). Aichi and poliovirus (PV) 3D^pol^ enzymes were incubated with LUVs in the presence of increasing concentrations of membrane-tethered 3B proteins, and the temporal fluorescence cross-correlation function between the CFP labeled 3D^pol^ and ATTO647N labeled LUVs was measured. In this analysis, the higher the cross-correlation the higher the binding (Fig. 4D, SI Fig. 4). We observed negligible binding of Aichi 3D^pol^ to neutral membranes at all 3B concentrations tested (0–2.7 µM) and small PV 3D^pol^ binding to neutral membranes at high membrane-tethered 3B concentrations. However, 3D^pol^ binding to acidic membranes (PI4P or PS) increased with increasing concentrations of membrane tethered 3B.

## Discussion

PI4P production is essential for many + RNA viruses, however, + RNA viruses do not encode PI4K enzymes and must hijack human enzymes. Many viruses including the Aichi virus converged on hijacking the PI4KB enzyme through the interaction with the ACBD3 protein. We recently showed that ACBD3 is a sub-micromolar binder of PI4KB and that ACBD3 can recruit the PI4KB enzyme to any membrane both *in vitro* and *in vivo* ^13^. Because the viral 3A protein is a transmembrane protein that tightly binds the GOLD domain of ACBD3^49^, it can recruit ACBD3 and thus the PI4KB to any target membrane. Soon after formation of the 3A:ACBD3:PI4KB protein complex, the membranes become hyper-phosphorylated, which is a key step in the biogenesis of replication organelles - a complex process that is not fully understood. It is initiated after polyprotein synthesis followed by stepwise proteolytic cleavage by viral proteases. These cleavage events produce intermediate as well as final cleavage products that together with host factors initiate replication complex formation. One of them is the 3A:ACBD3:PI4KB protein complex.

Here we reconstituted *in vitro* the formation of the 3A:ACBD3:PI4KB protein complex and membrane phosphorylation. We first prepared fluorescently labeled Aichi 3A protein and tethered it to the surface of GUVs to mimic infected cells (Fig. 1). As expected, the membrane-tethered 3A protein was able to recruit ACBD3 and subsequently the PI4KB kinase to the model membranes. Thus, our *in vitro* reconstitution experiments show that the small 3A protein is sufficient to recruit and activate PI4KB through interaction with ACBD3. This is an elegant mechanism – a protein less than 100 amino acids is all that is needed for the membrane hyper-phosphorylation – a key step of replication organelles formation. Congruently, when our manuscript was under review another study was published that also shows that viral 3A protein can activate PI4KB through the ACBD3 protein^50^.

The next key step in replication factory biogenesis is the recruitment of the viral 3D^pol^ enzyme. It was suggested that the 3D^pol^ enzyme binds directly to the PI4P lipid^39^. Therefore, we decided to reconstitute 3D^pol^ recruitment using our *in vitro* system. We did not observe 3D^pol^ recruitment to membranes under conditions in which the nanomolar PI4P reported was clearly recruited (SI Fig. 1). However, we did observe 3D^pol^ recruitment to PI4P rich membranes using a more sensitive technique (Fig. 4). Surprisingly, 3D^pol^ was also recruited to negative charged PS-containing membranes but not to neutral membranes (Fig. 4B). We repeated all experiments using the poliovirus 3D^pol^ enzyme to rule out the possibility that this mechanism is specific for the Aichi 3D^pol^ enzyme. Similar results were obtained, indicating that the 3B protein is crucial in recruitment of the 3D^pol^ enzymes. The PV 3B protein could somewhat recruit to neutral membranes while the Aichi 3B protein could not, but recruitment was more efficient in the context of negatively charged membranes. It is also worth mentioning, that we observed a somewhat lower cooperativity effect of the poliovirus 3B and 3D^pol^ proteins at the negatively charged membranes as documented by the lower amplitude of the cross-correlation function (SI Fig. 3). However, poliovirus 3D^pol^ is known to be able to form multimers^57,58^ and the avidity effect probably more than compensates. Interestingly, the PS lipid was significantly better in recruitment of the Aichi 3D^pol^ enzyme while in the case of the PV 3D^pol^ PS performed only slightly better than PI4P (Fig. 4D) which further supports our hypothesis that PI4P is not specifically recognized by picornaviral 3D^pol^ enzymes.

Many human and animal +RNA viruses including poliovirus (PV), Coxsackievirus B3 (CVB3), rhinovirus, norovirus, and HCV target the ER, Golgi or TGN membranes highly enriched in PI4P and cholesterol^59^. However, the observed interaction of 3D^pol^ enzymes with a properly (negatively) charged cholesterol rich model membrane is rather weak. Importantly, 3D^pol^ is a part of a relatively stable precursor protein that could affect its membrane recruitment in infected cells. The 3 CD^pro^ precursor protein of poliovirus is stable enough to be analyzed by protein crystallography^60^. The putative function of 3D^pol^ in the 3 CD^pro^ protein is to modulate the proteolytic activity of the 3C protease domain and RNA binding of the 3CD^pro^ protein^32^. It is important to emphasize that the picornaviral 3CD^pro^ has no polymerase activity. It only becomes active upon proteolytic cleavage of the link between the 3C and 3D proteins^32^.

**Figure 5:**
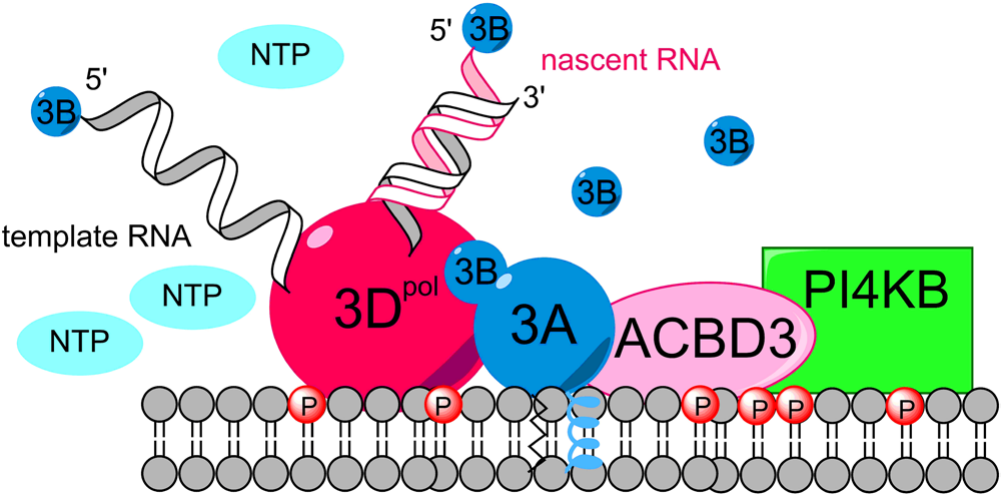
Schematic model of the 3D^pol^:3AB:ACBD3:PI4KB protein complex.

As with most polymerases, 3D^pol^ requires a primer. While *in vitro* it can initiate RNA synthesis using an RNA or DNA primer *in vivo* the viral 3B protein is always used as a primer and the first step is uridylylation of its tyrosine side chain^61^. The viral 3B protein is partially present as a membrane-tethered 3AB fusion protein and it was previously suggested to serve as a tether for 3D^pol^ *in vivo* ^28,62^. In our *in vitro* system, the membrane tethered 3B protein increased the efficiency of 3D^pol^ recruitment (Fig. 4D, SI Fig. 4). We conclude that negative charge and the membrane-tethered 3B protein work in combination to recruit 3D^pol^ enzymes. We thus propose that two protein complexes-3A:ACBD3:PI4KB (Fig. 2D) and 3D^pol^:3AB:ACBD3:PI4KB (Fig. 5) - are in a dynamic equilibrium in infected cells. While both are PI-phosphorylation competent only the latter also participates in RNA synthesis.

### Concluding Remarks

*In vitro* reconstitution is a powerful tool for testing biological hypotheses. The hypothesis that the non-structural 3A protein is the only viral protein needed to hyper-phosphorylate membranes when the cellular co-factors are present was proven in our *in vitro* system. However, our bio-mimetic GUV system reconstitution revealed that negative charged lipids and not necessarily PI4P are responsible for recruitment of the 3D^pol^ enzyme to the surface of the lipid bilayer. Moreover, our studies also revealed that the 3B protein works in combination with the negative charge to efficiently recruit the 3D^pol^ enzyme.

## Materials and Methods

### Cloning, Protein Expression and Purification

ACBD3, PI4KB (with deleted disordered loop 424–521 to increase stability and to facilitate bacterial expression), and mCherry-SidC (PI4P binding domains only, amino acid residues 613–742) were expressed as described previously^15^. AiV, EV 71, CVB3 3D^pol^ were cloned into the pRSFD vector with a His_6_-GB1 purification/solubility tag at the N-terminus followed by a TEV protease cleavage site. PV 3D^pol^ was expressed from the plasmid pCG1. This plasmid contains a ubiquitin tag at the N-terminus and His_6_ purification tag at the C-terminus of the PV 3D^pol^. To remove the ubiquitin tag and generate PV 3D^pol^ with a native N-terminus, the PV 3D^pol^ was co-expressed with ubiquitin protease in *E. coli* and further purified as previously described^63^. CFP-3A protein was cloned into a pRSFD vector with a His_6_ purification tag at the N-terminus followed by a TEV cleavage site and a mCerulean fluorescent protein (CFP) to produce final protein His_6_-TEV-CFP-His-Aichi 3A (residues 1–58)-His_6_. CFP-3D^pol^ proteins were cloned into pRSFD vector with a His_6_ purification tag at the N-terminus followed by a TEV cleavage site and mCerulean fluorescent protein to produce the final protein His_6_-TEV-CFP-3D^pol^. These proteins were purified using standard protocols in our laboratory^64,65^. Briefly, the proteins were expressed in BL21 Stars cells grown in autoinduction media at 18 °C overnight. Cells were lysed in lysis buffer (50 mM Tris pH 8, 300 mM NaCl, 3 mM βME, 10% glycerol) and nickel affinity purification was carried out according to the manufacturer’s instructions (Machery-Nagel). The His_6_ and His_6_-GB1 tags were removed by the TEV protease. The CFP-3A protein was prone to degradation, however, degradation products were removed by anion exchange chromatography. Finally, proteins were purified by size exclusion chromatography (SEC) in SEC buffer (20 mM Tris pH 8, 300 mM NaCl, 3 mM βME or 0.2 mM TCEP if the protein was meant to be labeled), concentrated to 3–10 mg/ml, aliquoted and stored at −80 °C until needed.

### Protein labeling with fluorescent dyes

ACBD3, 3D^pol^ and PI4KB were labeled on native cysteine residues as described previously^13^. Briefly, a 3-fold molar excess of rhodamine or Alexa488 maleimide derivatives (Molecular Probes) was incubated with pure recombinant proteins at 4 °C overnight. The reaction was quenched by adding a large molar excess of βME and the proteins were purified again on SEC. Labeling efficiency was estimated spectroscopically to be 116% for PI4KB labeled with Alexa488, 250% for 3D^pol^ labeled with rhodamine and 181% for ACBD3 labeled with rhodamine.

### Giant Unilamellar Vesicle Preparation and Imaging

Giant Unilamellar Vesicles (GUVs) were composed of POPC (54.9 mol %), POPS (5 or 10 mol %), PI4P (0 or 5 mol %) cholesterol (20 mol %), PI (10 mol %), DGS-NTA(Ni) (0 or 5 mol %) (Avanti Polar lipids), and ATTO647N-DOPE (0.1 mol %). Membrane composition for each experiment is also summarized in SI Table 1. When changing the concentration of the lipid mixture (e.g. to contain or not to contain PI4P or DGS-NTA(Ni)), charged lipids were always replaced with charged lipids (POPS for PI4P) and non-polar lipids with non-polar lipids (DGS-NTA(Ni) for POPC) to keep the net charge the same throughout all the experiments. GUVs were prepared by electroformation in a homemade teflon chamber as described previously^66^. Briefly, 50 µg of the lipid mixture in 10 µl volume was spread onto each ITO coated glass (5 × 5 cm) and dried under vacuum overnight. Later the glasses were placed in the teflon chamber separated by 1 mm thick spacers and 5 ml of 600 mM sucrose preheated to 60° was added and AC current (10 Hz, maximum amplitude 1 V) was applied for one hour in an incubator warmed to 60°. Then the chamber was allowed to cool down to room temperature and the GUVs were transferred with a glass pipette to a glass test tube. For imaging 100 µl of the GUVs were mixed with 100 µl of isotonic buffer (90 mM Tris pH 8, 20 mM MgCl_2_, 40 mM Imidazole, 565 mM NaCl, 3 mM βME) in a BSA coated 4-chamber glass bottom dish (*In Vitro* Scientific). GUVs were imaged using the filterless Zeiss LSM780 confocal system. To avoid bleed-through only mCerulean (CFP) tagged proteins (excitation 405 nm, emission 465–571 nm) and ATTO647-tagged lipids (excitation 633 nm, emission 645–759 nm) were excited simultaneously. Alexa488 (excitation 488, emission 508–578 nm), Rhodamine (excitation 561 nm, emission 565–585 nm) and mCherry (excitation 561 nm, emission 604–621 nm) fluorophores were excited separately. Images were processed using the ZEN 2012 software (Zeiss) and ImageJ^67^.

### Liposome (MLVs and LUVs) preparation

To prepare MLVs (multilamellar large vesicles) 1 mg of lipids composed of POPC (60 mol %), POPS (10 mol %), PI4P (10 mol %), and cholesterol (20 mol %) was mixed in chloroform. The chloroform was evaporated over a stream of dry nitrogen and the lipids were further dried under vacuum for at least three hours. Then the lipid film was rehydrated with 1 mL of liposome buffer (10 mM Tris pH = 7.4, 10 mM MgCl_2_, 150 mM NaCl, 3 mM βME) and vortexed intensively. To obtain LUVs (large unilamellar vesicles) the turbid solution containing MLVs was extruded 21 times using 100 nm filters in the extruder (Avanti Polar Lipids Inc, Alabaster, AL, U.S.A.).

### Liposome pulldown assay

Liposome pulldown assays were performed as described previously^68^. Briefly, MLVs with and without PI4P were prepared as described above. The liposomes were incubated with: AiV, PV, CVB3 and EV71 3D^pol^ enzymes (final protein concentration 60 μM and final lipid concentration 0.5 mg/ml) in a total volume of 60 μl. SidC (KD towards PI4P = 70 nM^38^) was used as a positive control (15 μM) and 14-3-3ζ (30 μM) was used as a negative control. The reaction mixtures were incubated for 20 min on ice. The mixtures were then centrifuged (22000 g, 10 min). The supernatant was removed and the pellet re-suspended in 60 μl of liposome buffer (20 mM Tris pH 7.4, 150 mM NaCl, 3 mM βME) and analyzed by SDS-PAGE. Control liposomes with no PI4P were used to confirm that binding was due to PI4P.

### Fluorescence cross-correlation spectroscopy (FCCS)

The FCCS experiments were carried out at LSM 780 confocal microscope (Zeiss, Jena, Germany) equipped with an LSM upgrade kit (Picoquant, Berlin, Germany) enabling Time-Correlated Single Photon Counting (TCSPC) acquisition. CFP and ATTO647N was excited by a 405 nm laser at 20 MHz repetition frequency, and by a 633 nm continuous-wave laser, respectively. A 40x/1.2 water objective was used together with a pinhole in the detection plain. Behind the pinhole, light was guided to the detectors (tau-SPADs, Picoquant), in front of which the emission filters (482/35, and 650/50) were placed. The collected data were correlated using a home-written script in Matlab (Mathworks, Natick, MA) according to the algorithm described in^69^. To avoid detector crosstalk, the red channel fluorescence signal was split according to its TCSPC pattern (exponential for the signal generated by the pulsed laser and flat for the 633 nm continuous wave laser) into two contributions and only the signal assigned to the flat TCSPC profile was correlated. Details of data processing are given in Gregor and Enderlein^70^.

50 µL of LUVs were mixed with 1 µL of CFP-3D^pol^ (100 nM), 0–20 µL of 3B-His (18 µM), and the LUV buffer, so that the final volume was 100 µL, the concentration of CFP-3D^pol^ was 1 nM, and the concentration of 3B-His ranged from 0 to 2.7 µM. The FCS experiment was carried out 15–20 minutes after mixing.

The lipid composition of LUVs is summarized in SI Table 1. Briefly: (POPC (64.99-x-y %), POPS (x%), PI4P (y%), PI (10%), cholesterol (20%), DGS-NTA(Ni) (5%), ATTO647N-DOPE (0.01%), where x = 0 and y = 0 for neutral membranes, x = 10 and y = 5 for acidic, PI4P-containing membranes, and x = 25% and y = 0% for acidic, highly charged membranes.

## Electronic supplementary material

Supplementary information
