# Supplementary material for: Negative charge and membrane-tethered viral 3B cooperate to recruit viral RNA dependent RNA polymerase 3Dpol
Source: Sci Rep. 2017 Dec 11;7:17309. doi: 10.1038/s41598-017-17621-6 (PMC5725453; doi:10.1038/s41598-017-17621-6)
Supplement: Supplementary file 1 — Supplementary information [file 41598_2017_17621_MOESM1_ESM.pdf]

Negative charge and membrane-tethered viral 3B cooperate to recruit viral RNA dependent RNA polymerase 3D^{pol}

Anna Dubankova[1,‡], Jana Humpolickova[1,‡], Martin Klima[1], Evzen Boura[1,*]

[1]Institute of Organic Chemistry and Biochemistry AS CR, v.v.i., Flemingovo nam. 2., 166 10 Prague 6, Czech Republic

[‡]these authors contributed equally

*correspondence to boura@uochb.cas.cz

Supplementary Figures

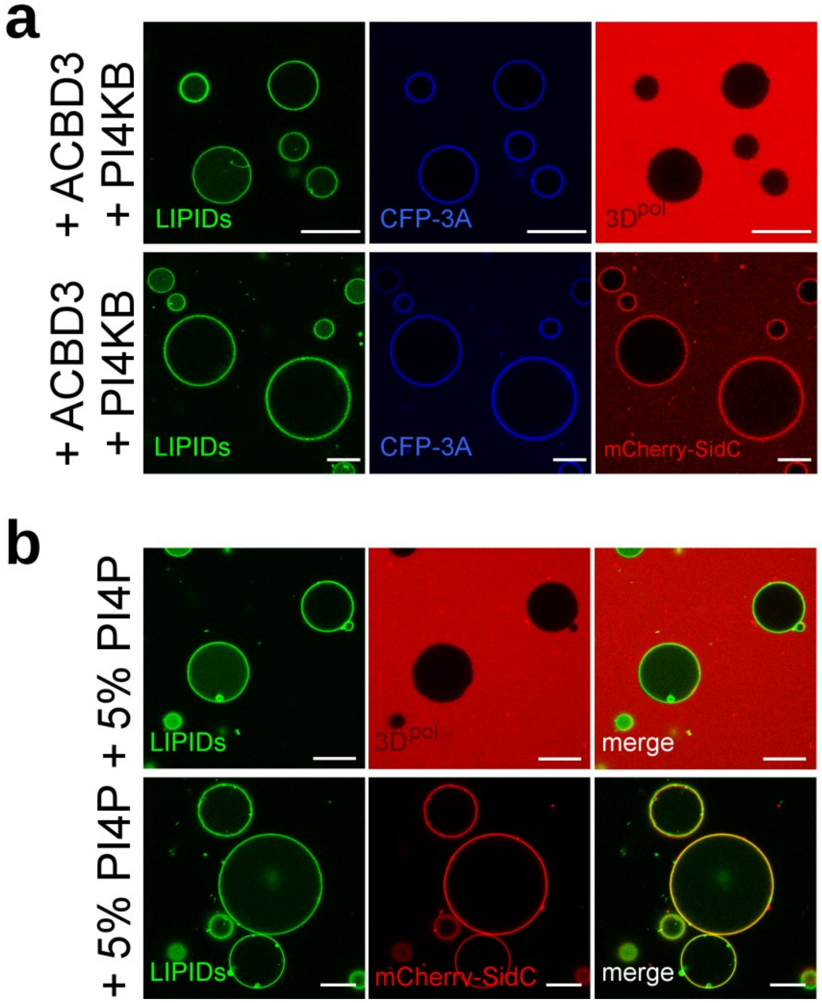

SI Figure 1 – PI4P mediated recruitment of 3D^{pol} under conditions where the nanomolar PI4P binder is recruited

A) **No recruitment observed upon membrane phosphorylation by the 3A:ACBD3:PI4KB protein complex.** Upper panel: GUVs were incubated with 250 nM 3A-CFP and PI4KB, 1 μM ACBD3 and 1 μM 3D^{po}l labeled by rhodamine. Lower panel: the same except that 3D^{pol} was replaced with 250 nM mCherry-SidC. GUVs contained 0.1% of PE-Fluorescein lipid (Avanti). Lipids are in green, CFP-3A is in blue, 3D^{pol} labeled by rhodamine or mCherry-SidC is in red. Representative image of two independent experiments. Scale bar = 20 μm.

B) **No recruitment observed using GUVs containing 5 mol % of synthetic PI4P.** Upper panel: GUVs were incubated with 1 μM 3D^{pol} labeled by rhodamine. Lower panel: the same except that 3D^{pol} was replaced with 250 nM mCherry-SidC. GUVs contained 0.1% of PE-Fluorescein lipid (Avanti). Lipids are in green, CFP-3A is in blue, 3D^{pol} labeled by rhodamine or mCherry-SidC is in red. Representative image of two independent experiments. Scale bar = 20 μm.

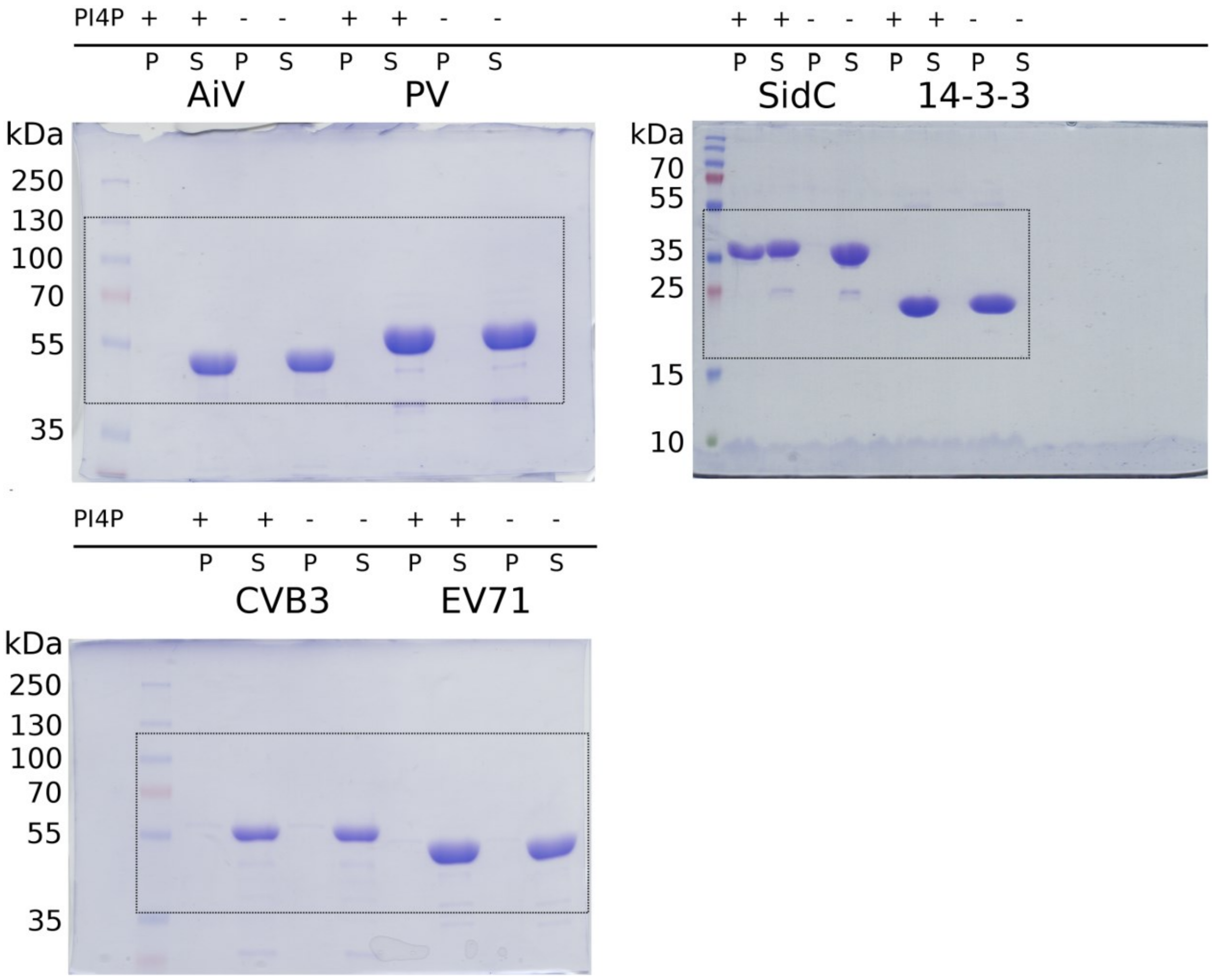

SI Figure 2 – Full length gels – Full length gel accompanying Figure 4A – Dashed rectangles are shown in Fig. 4

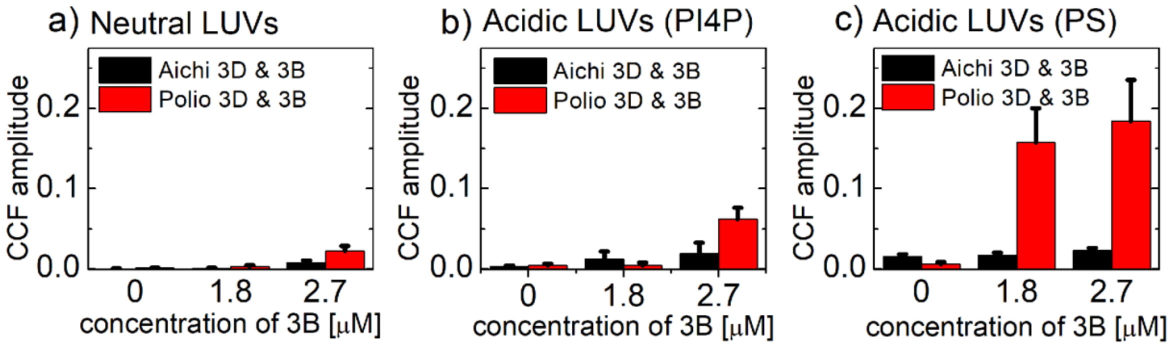

SI Figure 3 – Amplitudes of the functions that cross-correlate fluorescence signal of CFP-3D^{pol} and of LUVs labeled with ATTO647-DOPE. The amplitudes are shown for various LUV lipid compositions: a) neutral LUVs, b) acidic LUVs (PI4P), and c) acidic LUVs (PS), and for increasing amounts of membrane-tethered 3B. Black and red columns stand for Aichi and PV viral proteins, respectively. The error bar represents the standard error of the mean.

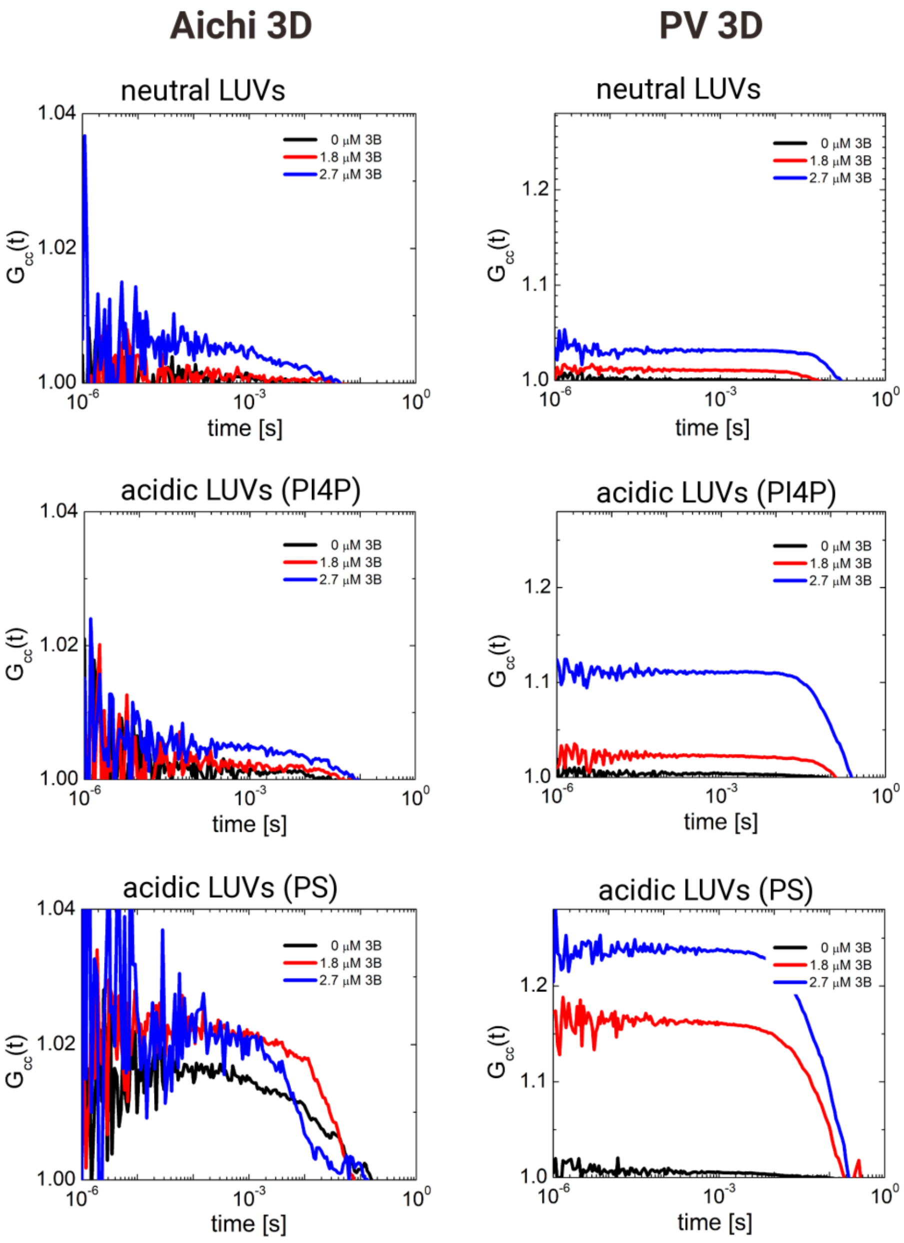

SI Figure 4 – An independent set of experiments conducted in the same way as in Figure 4D (Cros-scorrelation curves of CFP – 3D^{pol} and LUVs). FCS curves representing temporal cross-correlation of the CFP-3D^{pol} (Aichi 3D^{pol} on the left panel and PV 3D^{pol} on the right panel) and ATTO647N-DOPE labelled LUVs at various membrane lipid compositions (neutral, enriched by PI4P, and enriched by PS; see SI Table 1). The curves show the cooperative effect of the 3B peptide on 3D^{pol} membrane recruitment. The concentration of the Aichi or PV 3B peptide (attached to the membrane surface by His-tag – 18:1 DGS-NTA(Ni) interaction) was 0 μM (black), 1.8 μM (red), and 2.7 μM (blue).

Figure	Membrane type	POPC [%]	POPS [%]	PI [%]	PI4P [%]	cholesterol	DGS-NTA(Ni) [%]	Atto647N-DOPE [%]
1	GUVs	54.9	10	10	0	20	5	0.1
2	GUVs	54.9	10	10	0	20	5	0.1
3	GUVs	54.9	10	10	0	20	5	0.1
SI 1a	GUVs	54.9	10	10	0	20	5	0.1
SI 1b	GUVs	54.9	5	10	5	20	5	0.1
4b neutral,4c	GUVs	59.99	5	10	0	20	5	0.01
4b PI4P, 4c	GUVs	54.99	5	10	5	20	5	0.01
4b PS, 4c	GUVs	39.99	25	10	0	20	5	0.01
4a - PI4P	LUVs	60	20	0	0	20	0	0
4a + PI4P	LUVs	60	10	0	10	20	0	0
4d neutral	LUVs	64.99	0	10	0	20	5	0.01
4d acidic (PI4P)	LUVs	49.99	10	10	5	20	5	0.01
4d acidic (PS)	LUVs	39.99	25	10	0	20	5	0.01

SI Table 1: Membrane composition for each experiment.
